# Genetic and morphological shifts associated with climate change in a migratory bird

**DOI:** 10.1186/s12915-024-02107-5

**Published:** 2025-01-07

**Authors:** Nicole Adams, Tiffany Dias, Heather R. Skeen, Teresa Pegan, David E. Willard, Ben Winger, Kristen Ruegg, Brian C. Weeks, Rachael Bay

**Affiliations:** 1 Department of Evolution and Ecology, University of California Davis, Davis, CA 95616 USA; 2 Department of Ecology and Evolutionary Biology and Museum of Zoology, University of Michigan, Ann Arbor, MI 48109 USA; 3 School for Environment and Sustainability, University of Michigan, Ann Arbor, MI 48109 USA; 4 Negaunee Integrative Research Center, Field Museum of Natural History, Chicago, IL 60605 USA; 5 Department of Molecular and Cell Biology, University of Connecticut, Storrs, CT 06269 USA; 6 Museum of Zoology, University of Michigan, Ann Arbor, MI 48109 USA; 7 Department of Organismic and Evolutionary Biology, Harvard University, Cambridge, MA 02138 USA; 8 Gantz Family Collection Center, Field Museum of Natural History, Chicago, IL 60605 USA; 9 Department of Biology, Colorado State University, Fort Collins, CO 80523 USA

**Keywords:** Adaptation, Global change, Genomics, GWAS

## Abstract

**Background:**

Rapid morphological change is emerging as a consequence of climate change in many systems. It is intuitive to hypothesize that temporal morphological trends are driven by the same selective pressures that have established well-known ecogeographic patterns over spatial environmental gradients (e.g., Bergman’s and Allen’s rules). However, mechanistic understanding of contemporary morphological shifts is lacking.

**Results:**

We combine morphological data and whole genome sequencing from a four-decade dataset in the migratory bird hermit thrush (*Catharus guttatus*) to test whether morphological shifts over time are accompanied by genetic change. Using genome-wide association, we identify alleles associated with body size, bill length, and wing length. Shifts in morphology and concordant shifts in morphology-associated alleles over time would support a genetic basis for the observed changes in morphology over recent decades, potentially an adaptive response to climate change. In our data, bill size decreases were paralleled by genetic shifts in bill size-associated alleles. On the other hand, alleles associated with body size showed no shift in frequency over time.

**Conclusions:**

Together, our results show mixed support for evolutionary explanations of morphological response to climate change. Temporal shifts in alleles associated with bill size support the hypothesis that selection is driving temporal morphological trends. The lack of evidence for genetic shifts in body size alleles could be explained by a large role of plasticity or technical limitations associated with the likely polygenic architecture of body size, or both. Disentangling the mechanisms responsible for observed morphological response to changing environments will be vital for predicting future organismal and population responses to climate change.

**Supplementary Information:**

The online version contains supplementary material available at 10.1186/s12915-024-02107-5.

## Background

Climate influences every level of biodiversity, from the physiological performance of individuals to emergent properties of ecosystems [1]. Recent concern over anthropogenic climate change has led to an explosion of research linking climate to biological patterns and processes. In the evolutionary literature, there has been a strong focus on rapid adaptation as a potential mechanism allowing for population persistence [2–4]. Although methods and frameworks for predicting (mal)adaptation under future climate-induced selection pressures are gaining traction, in many wild populations, we lack an understanding of the rates, limits, and mechanisms of climate adaptation [5–7].

Interest in linking patterns of biodiversity at various levels of organization to climate, however, is far from new. For example, ecogeographic principles described over a century ago aimed to predict how species vary across global climate gradients, particularly across latitude. Bergmann’s Rule and Allen’s Rule invoke thermoregulatory principles to explain why organisms at higher latitudes tend to be larger [8, 9] and have smaller limbs [10], attributing these patterns to varying selection for heat exchange with the environment. Although initially supported in endotherms, these rules seem to apply across much of the tree of life and can predict distributions and trait variation both among and within species [9, 11] (but see [12]). The patterns of morphology across spatial environmental gradients have spurred questions about whether these same relationships can predict temporal trends as anthropogenic warming becomes increasingly severe [11, 13–16].

Independent time series now support the expected decrease in body size over time as temperature warms [17–20]. However, this finding is not universal, as predicted patterns may be counteracted by non-climatic factors [21, 22]. For example, across 42 avian species in Tanzania body mass increased 4.1% over the last three decades, though the mechanism for this counterexample remains unclear [23]. Many studies also find increasing appendage size with warming, including tails, legs, and ears in mammals and bills and tarsi in birds (reviewed in [13]). Despite the widespread documentation of morphological shifts, it is far from resolved whether these changes are driven by adaptation or plasticity, as few studies documenting contemporary morphological shifts are able to explicitly test for adaptation [14, 24]. Gienapp and Meliä [25] found decreased body size in Siberian Jays (*Perisoreus infaustus*) across two decades. Although body size is highly heritable in that system, quantitative genetic methods suggested that the majority of the observed temporal change was plastic. Plasticity has been shown to have an effect on morphological traits like body size; a recent review found that warmer temperatures during endotherm development most often lead to decreases in body size [26]. Morphological trends across generations are likely a complex combination of sometimes competing plastic and adaptive processes [27]. Further investigation is needed to determine to what extent morphological changes seemingly concordant with ecogeographic principles reflect selection.

Avian systems have been powerful models for studying rapid temporal shifts in morphology due to their well-described species diversity, broad distributions, and, importantly, rich historical collections allowing for robust investigations of temporal change. In general, there is evidence that birds follow ecogeographic rules; two separate reviews found that 72–76% of bird species adhere to Bergmann’s rule, with smaller body size in warmer environments [9, 11, 16]. Accordingly, many studies representing broad geographic and taxonomic ranges have found decreases in body size over time in recent decades [17–20]. While the evidence for Allen’s rule is more mixed, still many studies document larger appendages in warmer regions and warmer years [28–30]. Particular attention has been paid to the role of bill size in thermoregulation; since bills serve as a major source of heat dissipation, we expect birds in warmer regions to have larger bills [29, 31]. Although there is strong support for this trend in some systems [29, 30], recent studies have suggested more complex patterns, potentially due to the interaction of humidity and temperature in shaping bill size [28, 32]. Still, temporal studies find evidence for increasing bill length as temperatures warm [29, 30], though others find decreasing bill length, potentially due to constraints posed by shrinking body size [33]. Similar to the case with body size, the vast majority of these studies do not attempt to document genetic change over time.

Advances in DNA sequencing technology have increased the availability of temporal genomic datasets, allowing for direct investigation of genetic shifts associated with climate change [34, 35]. In this study, we aim to determine whether genetic variation associated with morphological traits show predictable shifts over recent decades, potentially driven by rising temperatures. Previously, two studies [18, 33] analyzed a set of > 70,000 birds from 52 species collected over a 40-year span during migration through Chicago, IL, USA and measured by a single observer. These studies documented consistent decreases in body size and bill length and increases in wing length. From this dataset, we selected a single species, hermit thrush (*Catharus guttatus*), that showed a decrease in body size and bill length over time and for which there were abundant appropriately preserved samples across the 40-year period. We combine morphological data with whole genome sequencing to identify genetic variants associated with body size, wing size, and bill size. We then ask whether those genetic variants shift over time in a manner that would be predicted by climate change-induced selection on morphological traits.

## Results

**Fig. 1:**
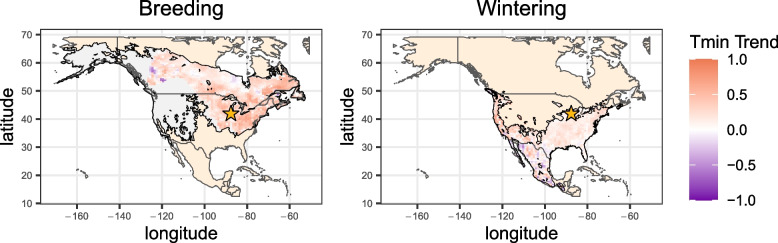
Trends in minimum temperature across the hermit thrush range. Colors reflect correlation (Spearman’s *⍴*) between minimum temperature and year for the breeding (left) and wintering (right) ranges. For the breeding range, trends are only shown for the East Taiga population [36], from which birds in our dataset originate (Additional file 1: Fig. S2). Gray regions represent the remainder of the breeding range of the species. Chicago, where birds for this study were collected during spring migration, is represented by a yellow star

Hermit thrush is a migratory species that breeds in coniferous boreal and montane forest habitats throughout the United States and Canada. In late fall, they migrate to wintering grounds in the southern United States, Mexico, and parts of Central America and return to the breeding grounds in spring. We visualized climate trends from 1981 to 2016, spanning the temporal range of our morphological dataset, in both the breeding and wintering ranges. The breeding range was cropped to include only the population likely to migrate through Chicago, where our samples were collected (the “East Taiga” population in Alvarado et al. [36]). Using rank tests, we focused primarily on the directionality of climate trends rather than significance. Across the 1981–2016 time period, most of the East Taiga breeding region experienced warming minimum temperatures, with 92.6% of grid points increasing over time (Fig. 1). Just over half (55.2%) of points showed increasing maximum temperatures (Additional file 1: Fig. S1) [36, 37]. The wintering range showed similar increases in minimum temperature, with warming at 84.1% of grid points, but geographic variation in maximum temperature trends, with 58.5% of points increasing. Together, this suggests that hermit thrush populations are experiencing warming minimum temperatures, with trends in maximum temperature more variable. In terms of precipitation, the East Taiga breeding region varied in response with about half (45.7%) experiencing drying while the majority (67.7%) of the wintering range experienced drying.

**Fig. 2:**
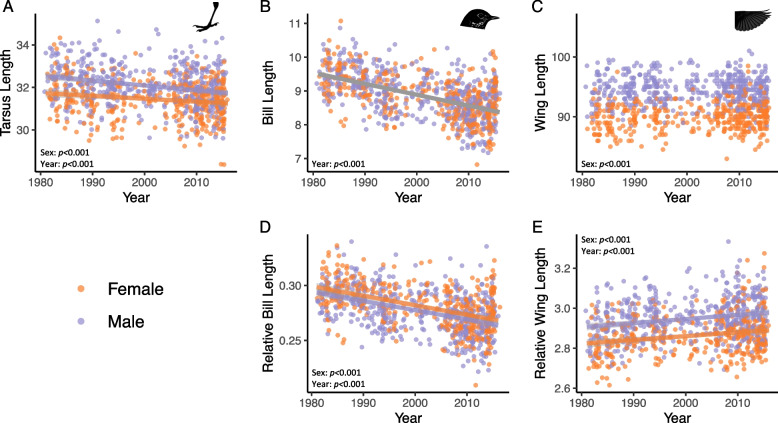
Morphological change in hermit thrush sampled over time. Plots reflect the effects of both sampling year and sex on trait measurements of tarsus length (**A**), bill length (**B**), and wing length (**C**) as well as bill length and wing length relative to tarsus (**D**, **E**). *p*-values are shown for significant terms under the best model, chosen based on AIC. Trendlines are shown for significant effects and separate trendlines for males and females are shown when sex was significant in the best model. Points are jittered to avoid overlap

Leveraging a prior morphological dataset of birds collected in Chicago, IL, USA during migration and measured by a single observer [18], we found shifts in body size, bill length, and wing length in hermit thrush over the last four decades (Fig. 2; Additional file 1: Table S1). For our study, we filtered this dataset to include only hermit thrush samples collected during spring migration (March–May), discarding hatch year birds and birds for which sex data were unavailable (*n* = 903 after filtering). Tarsus length, a common proxy for body size [38], decreased over time (Fig. 2A; *β* = − 0.018; *p* < 0.001). Absolute bill length decreased over time (*β* = − 0.032; *p* < 0.001), with birds collected in 2010–2015 having an average bill size 9.7% (0.9 mm) smaller than those collected in 1980–1985 (Fig. 2B). Relative bill length (i.e., scaled by tarsus length) also decreased over time (Fig. 2D; *β* = − 8.517e − 04; *p* < 0.001). While absolute wing length did not change over time (Fig. 2C), relative wing length increased over the past four decades (Fig. 2E; *β* = 0.002; *p* < 0.001). Males had longer tarsi and relative wing lengths, while females had slightly longer relative bill lengths. Taken together, these data show clear shifts in hermit thrush size and shape over the last 40 years.

We used whole genome sequencing of individuals collected during spring migration between 1986 and 2014 to investigate genetic shifts over time (Additional file 2: Table S2). Because population structure can confound GWAS results, we ran a joint analysis with previous genetic datasets from breeding season individuals (total *n* = 448) from across the entire breeding range [36, 37] to identify the breeding population for our samples. Population structure analysis in ADMIXTURE found four lineages (*K* = 4). As expected based on their sampling location in central North America, all individuals in our spring migration genetic dataset came from the Eastern Taiga breeding population, minimizing confounding effects of population structure on downstream GWAS (Additional file 1: Methods and Fig. S2).

Although hermit thrush are common, numbering over 70 million, they have experienced an estimated decline of ~ 9 million (~ 12%) in recent decades, which could result in loss of overall genetic diversity [39]. With the full set of ~ 24.5 million SNPs (single-nucleotide polymorphisms), across three different metrics, we did not find evidence for a temporal decrease in genetic diversity (Additional file 1: Fig. S3). Individual heterozygosity showed no significant trend over time (*p* = 0.21). Trends in θW and π were significant (*p* < 0.001), likely due to the large sample size achieved by sampling many genomic windows, but in both cases, the slope was positive (rather than the negative slope we would expect with loss of diversity over time), and the effect size was extremely small (θW: 8.9e − 6; π: 2.0e − 5). Together, this suggests no appreciable loss of genome-wide diversity in the last four decades.

Although genome-wide diversity was sustained, selection may result in allelic shifts at candidate loci encoding fitness-related traits. We used genome-wide association studies (GWAS) to identify allelic associations with morphology (for contemporary birds collected post-2005 only), with time, and with climate variables found in a previous study [18] to correlate with morphological change (breeding temperature, breeding precipitation, and nonbreeding precipitation). Based on an initial principal components analysis (PCA) showing separate clustering of males and females (Additional file 1: Fig. S4), we conducted separate GWAS for each sex. GWAS results, linked genes, and Gene Ontology (GO) terms can be found in Additional file 1: Figs S5-S9 and Additional file 2: Tables S3-S8. For tarsus length, our proxy for body size, candidate genes were associated with metabolic processes and anatomic structure development in males, but there were no significant GO terms for females. For wing length, there were no significant GO terms in males and genes linked to significant SNPs in females were enriched for a variety of GO terms associated with molecule localization.

**Fig. 3:**
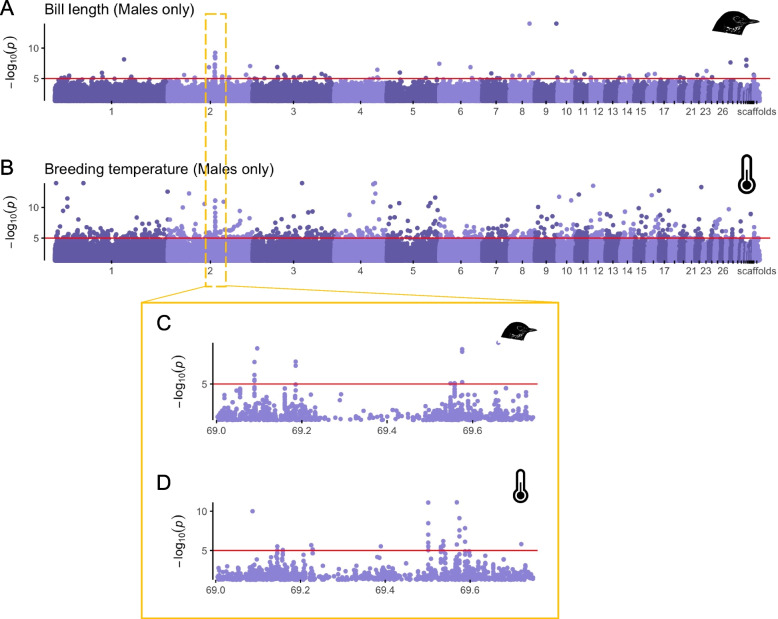
Genome-wide associations with bill length and breeding range temperature in hermit thrush males. Here, we show GWAS results genome wide for **A** bill length and **B** breeding range temperature. We also highlight the two peaks that make up the significant peak on chromosome 2 when examined more closely (**C**, **D**). The *x*-axis in **C** and **D** represents the position (in bp) along that chromosome. The red line represents a significance cutoff of *p* < 1e − 5

The strongest apparent GWAS peak was a region on chromosome 2 associated with bill length in contemporary males (Fig. 3A). Interestingly, this region was not associated with bill length in females (Additional file 1: Fig. S6). Upon further investigation, a likely explanation is that alternate allele frequencies in this region were lower in females (5–10%) compared to males (11–17%), so we do not have power to determine whether these alleles are also associated with bill length in females. A total of 86 SNPs were significantly associated with bill length in males and of these 9 are found in the chromosome 2 peak. Although 40 genes were linked to SNPs associated with bill size, no annotated genes were found in the peak on chromosome 2. Gene Ontology (GO) terms enriched for genes associated with bill length were related to nervous system functions, including behavior, transmembrane transport, and neuron development, potentially related to the neural crest origin of the avian bill. A similar though less significant peak also occurred for wing length (Additional file 1: Fig. S7). We also noted a GWAS peak in a similar location on chromosome 2 associated with climate, specifically breeding range temperature and wintering range precipitation (Fig. 3B; Additional file 1: Fig. S6). Zooming in on that region alone, we find the peak associated with bill size lies in two distinct regions (Fig. 3C) and that different SNPs within that same region comprise the peak associated with climate (Fig. 3D). Across all chromosomes, 293 genes were linked to 689 SNPs associated with breeding range temperature in males across the whole time series. These genes have functions including locomotion, brain development and behavior (among others).

**Fig. 4:**
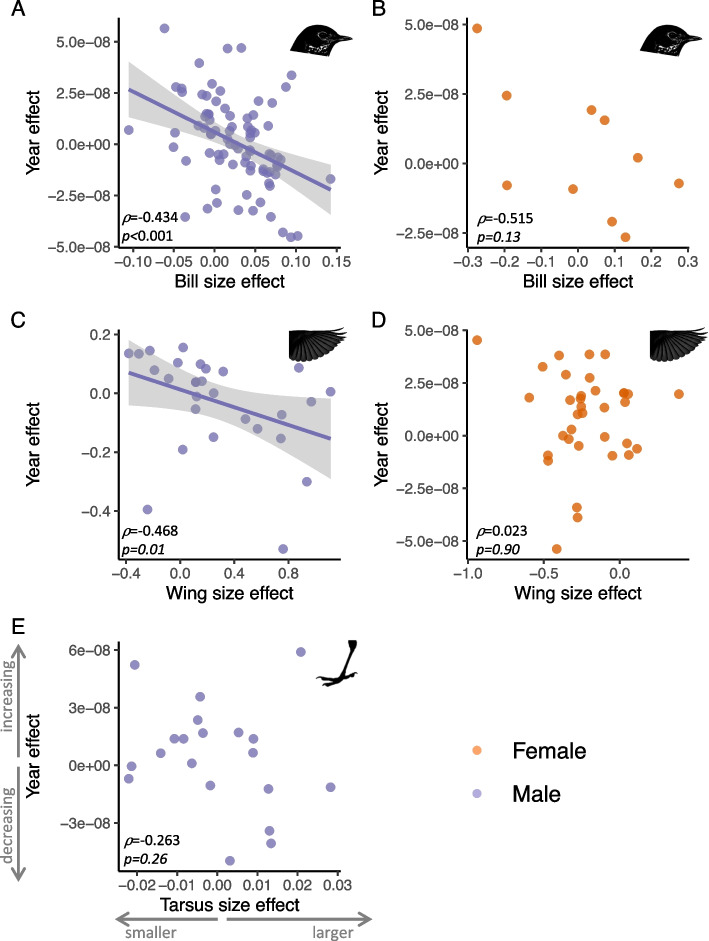
Shifts in frequencies of morphology-associated alleles over time. For GWAS candidate SNPs associated with morphology, we estimated effect sizes for morphology and year response variables. Plots are shown separately for bill length (**A**, **B**), wing length (**C**, **D**), and tarsus. SNP candidates were analyzed separately for males (left column) and females (right column). No plot was included for tarsus length in females because no significant SNPs were identified in the GWAS. A significant correlation means that SNPs most strongly associated with morphology also change most over time. Trend lines are included when the relationship is significant

We assessed whether morphology-associated SNPs identified by GWAS showed shifts in allele frequencies over time by comparing effect sizes when morphology was treated as the response variable to those with year as the response variable. Effects sizes (*β*) were calculated using ridge regression [40] and a correlation between effect sizes for morphology and time suggests that SNPs with relatively large effect on morphology also change most over time. Candidate SNPs associated with both bill length and wing length in contemporary males exhibited changes in allele frequency over time. Specifically, alleles associated with large bill sizes in males have decreased over time, as demonstrated by the negative correlation between effect sizes of candidate SNPs for explaining bill length (*β*_Bill_) and year (*β*_Year_) (Fig. 4A; Spearman’s *⍴* = − 0.434; *p* < 0.001). There is also a negative trend in females, though the effect is not significant (Fig. 4B; Spearman’s *⍴* = − 0.515; *p* = 0.13). Alleles associated with larger wings in males decreased over time (Fig. 4C; Spearman’s *⍴* = − 0.468; *p* = 0.01), although this contradicts our morphological analysis in which we did not document significant changes in absolute wing length and relative wing length shows the opposite trend, increasing over time. Effect size comparisons with relative bill and relative wing length were qualitatively similar (Additional file 1: Fig. S10). All other comparisons of morphological and temporal effect sizes were non-significant (Additional file 1: Table S9). Interestingly, effect sizes for males and females were not positively correlated for any morphological trait, suggesting that alleles with large effect in one sex did not have large effects in the other.

## Discussion

Multiple studies have now documented morphological shifts over time concordant with changing climate conditions. While often assumed to be adaptive, the role of genetics in such changes including the relative contributions of genetics and plasticity, the genetic architecture of the traits, and the limits to adaptation are relatively unknown [15, 24, 27, 41]. Here, we combine morphological and whole genome sequencing data to ask whether changes in body size and shape over the past four decades are accompanied by genetic shifts in hermit thrush. We find a decrease in body size (i.e., tarsus), but no corresponding temporal shifts in alleles associated with body size. We find a dramatic decrease in bill size, as observed in other species within the same dataset [33], and concordant shifts in bill-associated alleles over time. Together, our data suggest that some components of morphological change are accompanied by genetic shifts, consistent with selection driven by ongoing climate change.

Although we observe shifts in body size over time consistent with Bergmann’s rule, we do not observe allelic shifts supporting a genetic basis for this change. Our morphological data is a subset from a previous study that found consistent declines in body size across 52 bird species over 40 years [18]. These declines were correlated with temporal shifts in climate, particularly temperature in the breeding range [18]. In our subset data of hermit thrush spring migrants, we also see an overall decrease in body size. SNPs associated with body size in contemporary individuals were linked to 11 genes (10 in males, 1 in females). One of these (*BICC1*) was previously associated with growth in chickens [42, 43], and two (*TFAP2D, KIAA0556*) were associated with reproductive output in birds, which may also be linked to body size [44, 45]. However, we did not find temporal allele frequency shifts in body size-associated SNPs. Of course, a caveat to our study, and all genome scans in natural systems, is that sample size is limited and we likely overlook alleles with small to moderate effect sizes, especially with a polygenic trait like body size [46].

One potential biological explanation for the lack of temporal signal is that shifts in body size are not the result of selection, but plastic responses to environmental change. Although body size in many avian systems is highly heritable, temperature also has plastic effects on body size and these two forces can counteract one another [26, 27, 47]. A small number of previous studies using quantitative genetics to investigate shifts in body size have found similar results; despite changes in measured body size, breeding values did not show substantial shifts, suggesting selection is not responsible [25, 41]. On the other hand, most studies examining plastic effects of developmental temperature find that warming decreases body size, though this effect is neither universal nor linear and may depend both on the magnitude of warming and how far the temperature is from the optimal developmental temperature [26, 27]. Furthermore, the question of whether the relatively small thermoregulatory benefits of shrinking body size can sufficiently explain temporal trends is debated [48]. Plasticity could also be a potential explanation for the contradicting results we find for wing length; relative wing length increases over time while alleles associated with long wings decrease over time. Competing plastic responses and selective pressures can result in unpredictable morphological outcomes [27]. Further studies exploring how temperature shifts both plastic and genetic components of morphology will be required to gain an understanding of the costs and limits to morphological change.

We find a substantial decrease in bill size over time accompanied by genetic shifts. Bill size may be constrained by body size itself, the decrease could be due to selection on body size rather than direct selection on bill size. Previous analysis of the larger dataset of Chicago migrants found that across 52 species bill sizes decreased over time, and the rate of morphological change was dependent on body size, such that smaller species changed at a higher rate [33]. In the context of Allen’s rule, the direction of morphological change we observe is opposite of our expectation. Avian bills are vascularized structures and can be used for thermoregulation [31]. Following Allen’s rule, we would expect larger bills in warmer climates and an increase in bill size over time as temperatures warm. Both spatial and temporal trends have been seen in other systems. In California Savannah sparrows (*Passerculus sandwichensis*), for example, bills have higher surface area in hot dry habitats and within the coastal subspecies bill size has increased over the last century [29]. Recent work, however, has demonstrated that the climate drivers of bill size are more complex and bill size can be affected by both temperature means and extremes as well as precipitation [28, 32]. In fact, Subasinghe et al. [28], looking across 79 species of Australian passerines, showed that when rainfall exceeds ~ 100 mm, there was a negative correlation between bill size and maximum temperature, more similar to the pattern we see in our data. Additionally, other functions of bills like diet might result in contrasting selection pressures affecting bill size.

GO categories enriched for SNP associations with bill size were largely involved in development and nervous system processes. This potentially reflects the fact that bills are shaped from neural crest cells [49]. Although we did not observe SNPs associated with bone morphogenic protein *BMP4,* which has been linked to bill size in birds [50], genes involved in BMP regulation (*CRIM1*, *GCM2*) were included in our list of candidates [51, 52]. In contrast to body size, alleles associated with bill size changed over time, concordant with the observed morphological change. Specifically, alleles associated with larger bills decreased in frequency over time while alleles associated with smaller bills increased in frequency. However, as with all morphological traits in our analysis, effect sizes for male and female bill size were not positively correlated, suggesting that alleles with large effect in one sex did not have large effects in the other. This could have a number of either biological (e.g., sex-specific allelic effects) or technical (e.g., sample size) explanations. The combined decrease in size and shift in bill-associated alleles are consistent with selection for smaller bills over time, though the mechanism of selection is not clear.

## Conclusions

As we become more aware of the potential consequences of climate change on biodiversity, the availability of temporal datasets is increasing. In the past decade, a growing number of studies have shown morphological change associated with rapid temperature changes [13, 16–18, 20]. However, genetic investigation of the underlying mechanisms of these shifts is just beginning. Here, we show that genetic shifts can accompany rapid climate-driven shifts in morphology, but this is not universal. Likely morphological change is driven by the complex interactions between adaptation and plasticity. Elucidating the mechanisms driving this change will lead to better prediction of future population responses to global change and increase our understanding of rapid evolutionary processes more generally.

## Methods

### Range-wide climate trends

To visualize patterns of climate change experienced by hermit thrush populations in recent decades, we downloaded daily climate data for both the breeding and non-breeding ranges. Because our dataset consists of birds that migrate through Chicago, they are not drawn from the entire breeding range, but only the “East Taiga” population (defined by Alvarado et al. [36]; see “population assignment”). We therefore clipped the breeding range to only include this region, but we used the entire wintering range of the species. Within the range, we created a 1° longitude × 0.5° latitude grid. For each point in the grid, we downloaded daily climate data from the Daymet database [53] for all years spanning the range of our morphological samples (1981–2016). For each point within the breeding range, we calculated minimum temperature, maximum temperature, and total precipitation for the breeding months (June and July). Because minimum and maximum temperatures are subject to extreme values, we used instead the 95th (for maximum temperature) and or the 5th (for minimum temperature) quantile. We calculated these same climate variables in the non-breeding range for the winter months (January and February). Then, for each point and each climate variable, we calculated Spearman’s correlation coefficient (*⍴*) to estimate the trend over time.

### Morphological shifts

Morphology data for multiple bird species were collected over a nearly 40-year period (1978–2016) and measured by a single person (David E. Willard) as part of a previous study [18]. That study found a consistent signature of decreasing tarsus length, a proxy for body size, over time. For our study, we filtered this dataset to include only hermit thrush samples collected during spring migration, discarding hatch year birds (to avoid possible confounding variation in allometric relationships due to age) and birds for which sex data was unavailable. This left us with data for 903 birds spanning 1981–2016. To account for allometric scaling, we calculated relative bill and relative wing lengths by dividing by tarsus length. We reanalyzed this single species data set using linear regression to test for effects of year, sex, and the interaction between year and sex on each morphological trait (bill length, tarsus length, and wing length, relative bill length, relative wing length). We determined the best model by sequentially dropping terms and comparing AIC scores.

### Sampling and DNA extraction

From the filtered set of hermit thrushes collected during spring migration as part of the Weeks et al. [18] study, the Field Museum provided tissue samples (*N* = 7) and DNA extractions (*N* = 229) spanning 1986–2014. All birds selected for sequencing were after hatch year birds collected during spring migration. Whole genomic DNA was extracted from muscle tissue using modified protocols based on DNeasy Blood & Tissue Kits (Qiagen). The eluted DNA samples we received were extracted with the following modifications: pectoral muscle was extracted by digestion in 10 mM Tris, 2 mM EDTA, 1% SDS, and Proteinase K at 56 °C for 24 h. The DNA was then bound to silica in the presence of 6 M GuHCl, 3.75 M NH4Ac pH 6, and ethanol, washed twice with 10 mM Tris–HCL and 80% EtOH and eluted with ultrapure water or low TE (sensu [54]). For tissue samples, DNA was extracted following the DNeasy Blood & Tissue Kit protocol with these modifications: samples were incubated overnight at 56 °C, the sample was passed over the spin column twice prior to washing, an extra column drying step was taken (14,000 rpm for 3 min), and DNA was eluted in 200 μl AE buffer heated to 56 °C. AE buffer was incubated on the filter for 5 min instead of two in the final elution step. The elution was pipetted back onto the filter for a second pass through after incubating for another 5 min. Whole genomic DNA was quantified using a Qubit Fluorometer (Thermo Fisher Scientific), and the quality of DNA was assessed using a 2% agarose gel.

### Library preparation and whole genome sequencing

We used a modified library preparation based on Illumina’s Nextera protocol [55] to sequence entire genomes of 236 birds. To start, genomic DNA was standardized to 2.5 ng/μl then underwent a tagmentation step using TDE1 enzyme and buffer (Illumina). Dual combination Nextera indexes (Illumina) were then added to tagged DNA fragments followed by a booster PCR using the Kapa HiFi Kit (Kapa Biosystems). Libraries were then bead cleaned and double size selected to remove fragments > 700 bp and < 320 bp using AMPure XP Beads (Beckman Coulter) and quantified using a Qubit Fluorometer (Thermo Fisher Scientific). The 118 libraries with the greatest mass of DNA available were pooled equimolarly into a single pooled library (Library ID = 006, 33.29 ng DNA per sample), while the 118 libraries with lower masses of DNA available were pooled equimolarly into a separate library (Library ID = 005, 6.0 ng DNA per sample). Both pooled libraries were then visualized with a Bioanalyzer (Agilent). The pooled libraries were further size selected to remove residual fragments < 320 bp using a left side AMPure XP Bead cleanup. Each final pooled library was sequenced on a separate lane of NovaSeq 6000 as 150 bp paired-end reads to target 4X coverage, and the resulting sequences were demultiplexed by Novogene (Sacramento, CA, USA).

### Data processing

Adapters and low-quality reads were trimmed using Trim Galore! (a wrapper around Cutadapt [56], accessible at http://www.bioinformatics.babraham.ac.uk/projects/trim_galore). Each sample was aligned to the Swainson’s thrush (*Catharus ustulatus*) reference genome, GCA_009819885.2, using BWA-MEM [57] then sorted and indexed using Samtools [58]. We used the Swainson’s thrush reference genome (GCA_009819885.2) because there currently is not a chromosome-level, annotated genome for Hermit Thrush. Duplicate reads were marked with MarkDuplicates, and coverage was estimated using CollectWgsMetrics from Picard Tools (http://broadinstitute.github.io/picard). Since our data were low coverage, we identified single-nucleotide polymorphisms (SNPs) and estimated genotype likelihoods using the program ANGSD [59]. We created a BEAGLE file of genotype likelihoods (-doGLF 2) using the GATK method (-GL 2) for all individuals and removed reads that had multiple best mapping hits (-uniqueOnly 1), failed or were duplicates (-remove_bads 1), did not have a mapped mate (-only_proper_pairs 1), or had more than two alleles (-skipTriallelic 1). To produce a set of high-quality variants, we removed sites below a minimum base quality (-minQ 20) and mapping quality (-minMapQ 20), a minimum minor allele frequency (-minMaf 0.02), and a low maximum likelihood of being polymorphic (-SNP_pval 2e-6). Additionally, we removed potential paralogs (-doHWE 1 -maxHetFreq 0.5) and required sites to be found across a minimum of 197 individuals (-minInd 197). Sex chromosomes were removed for downstream processing. A BEAGLE file for contemporary samples, those collected 2005 or later, was made with the same ANGSD parameters, except a minimum of 131 individuals (-minInd 131) was used.

### Population assignment

Previous work found population structure across the North American breeding range for hermit thrush [36]. Genetic structure can confound GWAS results, so we wanted to avoid sampling across multiple populations. Since our samples were collected in Chicago during migration, they likely represent birds breeding in the single population that stretched from northern British Columbia to eastern United States and Canada, the Eastern Taiga population [36]. To verify that none of our samples belonged to other genetic groups, we conducted an analysis of population structure, combining our samples with those from two other projects. The first additional dataset was from a RADseq project that demonstrated the population structure across North America [36]. The second dataset consisted of raw low coverage whole genome fastq files from samples collected in the upper Midwest of the United States and central Canada [37] (Additional file 2: Table S2), regions missing from the Alvarado et al. [36] study. We processed the whole genome data as above with the exception of aligning to the draft hermit thrush genome from Alvarado et al. [36], which allowed us to directly use genotypes from that study. We removed individuals (*N* = 2) from the dataset that had low individual coverage (< 0.01X). Combining all three datasets, we had 448 samples from across the breeding range (Additional file 1: Fig. S2 A). We called SNPs with bcftools mpileup followed by bcftools call [60] at the same sites that were identified in the hermit thrush RADseq dataset. We then combined SNPs and filtered to retain only SNPs covered in all three datasets, which left us with 29,506 SNPs. We conducted a principal components analysis using the snpgdsPCA function in the SNPRelate R package [61]. Additionally, we used assignPOP [62] to assign samples from this study to genetic clusters identified by Alvarado et al. [36] using the other two datasets with known assignments. We ran the assign.X function with three different classification models: random forest, naive Bayes, and support vector machine. All methods produced the same general result so we only show the random forest results.

### Genetic diversity

To test for changes in genetic diversity over time, we estimated heterozygosity, Watterson’s theta (θW) [63], and nucleotide diversity (π) [64]. Heterozygosity was measured at the individual level as the proportion of heterozygous genotypes per sample. For each individual remaining after filtering for coverage and population identity, we used ANGSD to identify SNPs and estimate site allele frequencies (SAF) and realSFS to calculate the folded site frequency spectrum (SFS). We estimated the proportion of heterozygous sites from the SFS for each sample. For θW and π, we chose to calculate these metrics per year only for years represented by at least 5 samples. We first used ANGSD to estimate SAFs then realSFS to calculate the folded SFS for each group representing a single year. We then re-ran ANGSD to calculate diversity metrics (-doThetas 1) using the SFS as the prior (-pest). For each year group, we calculated diversity metrics in non-overlapping 10 kb sliding windows.

### GWAS

We used genome-wide association tests, implemented in ANGSD, to identify SNPs associated with three morphological measures: bill length, tarsus length, and wing length. For GWAS with morphological traits as the response variable, we used “contemporary” samples only, those collected after 2005, in an attempt to avoid confounding factors that might change over time. Our preliminary analysis showed that there were no significant time trends in these traits between 2005 and 2014. First, we conducted principal components analysis (PCA) in PCAngsd [65] to estimate PC axes which could be included as covariates in our GWAS to account for relatedness among samples. PCA was conducted with a subset of LD-thinned SNPs which were identified using PLINK (–ld-window-kb 500, –ld-window-r2 0.05) [66]. Since an initial PCA showed strong separation of sexes even when only autosomal SNPs were analyzed (Additional file 1: S4), we chose to conduct GWAS separately for males and females. We therefore recalculated the PCA with LD-thinned SNPs separately for males and females and used the first 10 PC axes of each, along with sequencing library ID and tarsus length (for bill and wing length only), as covariates for the morphological GWAS. GWAS were conducted in ANGSD (-doAsso 2, -minMaf 0.1). We used a significance cutoff of *p* < 1e-5 to identify candidate SNPs.

In addition to morphological GWAS conducted with contemporary samples only, we also used the GWAS framework to test for genotypic associations with both time (i.e., year) and three climate variables that were previously found to be associated with morphology: breeding temperature, breeding precipitation, and wintering temperature [18]. Climate variables for this analysis were taken from this previous study. These GWAS were performed similarly to those for morphological traits but included samples from all years and were performed for males and females separately. We included 10 PC axes and library ID as covariates.

Once we identified candidate SNPs associated with morphological traits, we tested for trends in allele frequencies at these SNPs over time using two separate methods. First, we calculated effect size estimates (*β*) at those SNP positions for association with the morphological trait (using just contemporary samples) and for association with year (using all samples). Hereafter, we use the *β* subscript to denote the response variable (e.g., *β*_Bill_ or *β*_Year_). A correlation between *β*_Morphology_ and *β*_Year_ would suggest that SNPs with large effects on morphology are also changing most over time and can provide information about the directionality of that change. Because the GWAS score test in ANGSD does not compute effect sizes, we used rrBLUP [40] to compute *β* using the mixed.solve() function. As input, we used estimated allelic dosage based on genotype probabilities output by ANGSD and computed using BEAGLE [67]. We then tested for correlations between the relevant *β*_Morphology_ and *β*_Year_ using a Spearman correlation test. Note that beta coefficients were calculated for a single sex. We separately calculated coefficients for the same SNPs on the opposite sex to test whether variation at these SNPs had power in the other sex. For example, for candidate SNPs associated with bill size in males only, we calculated *β*_Bill_ for males only, *β*_Year_ for males only, and *β*_Bill_ for females only. The comparison of *β*_Bill_ to *β*_Year_ in males tests whether SNPs associated with bill size in contemporary male samples vary over time in males. The comparison of *β*_Bill_ in males to β_Bill_ in females tests whether SNPs associated with bill size in contemporary males also explain variation in contemporary female bill size. For GWAS candidates for bill and wing, we ran this analysis using both absolute and relative morphological measurements.

### Annotation

For each set of candidate SNPs derived from GWAS, we created a list of linked genes. We used LD-annot [68], which finds genes in linkage disequilibrium (*r* > 0.9) with candidate SNPs. We used these gene lists to test for enrichment of gene ontology (GO) terms. Because annotation of the current version of the Swainson’s thrush genome does not contain GO terms, we extracted GO terms from the zebra finch and chicken genomes based on matching gene names in biomaRt [69]. We then used topGO [70], which employs a Fisher’s exact test to test for overrepresentation of GO categories in a particular candidate set, given a background of all genes linked to all SNPs.

## Supplementary Information

Additional file 1: Figs. S1-10, Tables S1,S9. Fig. S1. Climate trends across the breeding and wintering ranges. Fig. S2. Population assignment of spring migrants. Fig. S3. Genomic diversity over time. Fig. S4. PCA across all samples. Fig. S5. Body size GWAS. Fig. S6. Bill length GWAS. Fig. S7. Wing length GWAS. Fig. S8. Climate variable GWAS in males. Fig. S9. Climate variable GWAS in females. Fig. S10. Correlation between year and relative morphology effect size. Table S1. Effects of year and sex on morphological traits. Table S9. Shifts in GWAS candidate SNPs over time.

Additional file 2: Tables S2-S8. Table S2. Specimen metadata. Table S3. Genes associated with morphology in males. Table S4. Genes associate with year or climate in males. Table S5. Genes associated with morphology in females. Table S6. Genes associated with year or climate in females. Table S7. GO term enrichment in males. Table S8. GO term enrichment in females.

## Data Availability

Raw data for newly sequenced samples are deposited in NCBI SRA (PRJNA1135140). Metadata is available in Additional file 2: Table S2 for newly sequenced samples and samples used from [37]. Code used for bioinformatics and data analysis is available at https://github.com/rachaelbay/HETHClimate.

## Abbreviations

SNP: Single-nucleotide polymorphism
GWAS: Genome-wide association study
PCA: Principal components analysis
GO: Gene ontology
