# Supplementary material for: Genetic and morphological shifts associated with climate change in a migratory bird
Source: BMC Biol. 2025 Jan 7;23:3. doi: 10.1186/s12915-024-02107-5 (PMC11705884; doi:10.1186/s12915-024-02107-5)
Supplement: Supplementary file 1 — Additional file 1: Figs. S1-10, Tables S1,S9. Fig. S1. Climate trends across the breeding and wintering ranges. Fig. S2. Population assignment of spring migrants. Fig. S3. Genomic diversity over time. Fig. S4. PCA across all samples. Fig. S5. Body size GWAS. Fig. S6. Bill length GWAS. Fig. S7. Wing length GWAS. Fig. S8. Climate variable GWAS in males. Fig. S9. Climate variable GWAS in females. Fig. S10. Correlation between year and relative morphology effect size. Table S1. Effects of year and sex on morphological traits. Table S9. Shifts in GWAS candidate SNPs over time. [file 12915_2024_2107_MOESM1_ESM.docx]

**Figure S1.** Climate trends across the breeding (A-F) and wintering (G-L) ranges for Hermit Thrush. Colors show the magnitude and direction of change for minimum temperature (A,D,G,J), maximum temperature (B,E,H,K), and precipitation (C,F,I,L) from 1986-2014. Trends were calculated using a Spearman’s rank test. Gray region in A-C represents the breeding range outside of the East Taiga population.


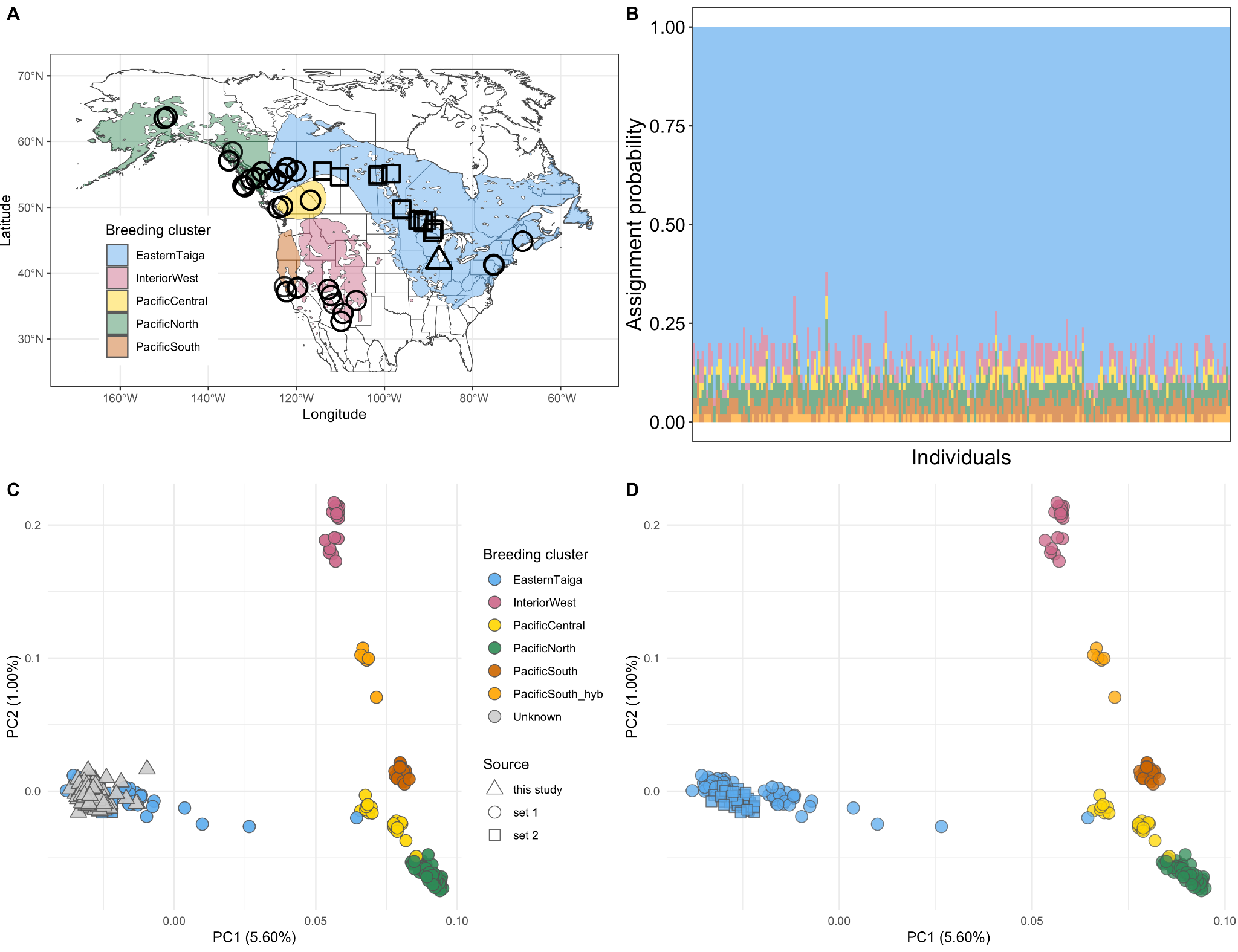


**Figure S2.** Assignment of individuals collected during spring migration to breeding populations. A) Map showing Hermit Thrush breeding populations (defined by Alvarado et al. 2022) along with the locations of collection for samples from our study. B) random forest population assignment analysis of spring migration samples collected in Chicago to breeding clusters. C,D) PCA of individuals collected across the range during breeding season. In (C) we display all individuals, while in (D) we remove individuals caught during spring migration to show the cluster of breeding season individuals underneath. The breeding range samples used for population assignment included samples from this study, the RADseq study (set 1: Alvarado et al. 2022), and the low coverage whole genome study (set 2: Pegan et al. 2024). The analyses are based on 29,506 SNPs.

**
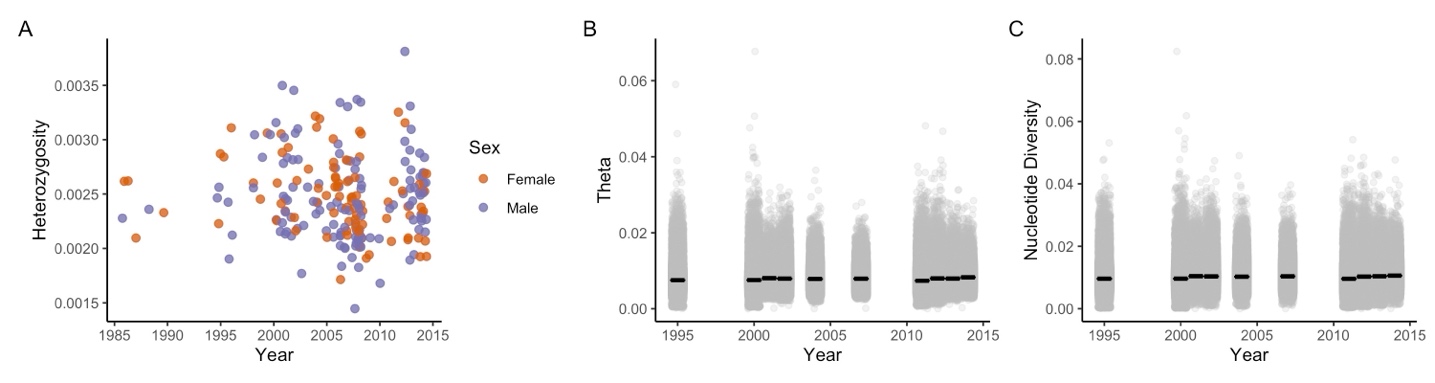
**

**Figure S3.** Genomic diversity over time, measured as individual heterozygosity (A), Watterson’s theta (B), and Nucleotide Diversity (C). Theta and nucleotide diversity are measured in 10kb windows and only for years represented by at least five individuals. None of the measures of genomic diversity show decreases over time.

**Figure S4.** Principal components plot created using 2,446,336 SNPs across all samples, pruned based on linkage disequilibrium.

**Figure S5**. Genome-wide associations with body size (i.e. tarsus length) for males (A,B) and females (C,D). For morphological GWAS only contemporary (post 2005) birds were considered. Red lines in A & C represent a significance cutoff of *p*<1e-5. Plots B & D are Q-Q plots with the red line representing the null expectation (1:1).

**Figure S6**. Genome-wide associations with bill length for males (A,B) and females (C,D). For morphological GWAS only contemporary (post 2005) birds were considered. Red lines in A & C represent a significance cutoff of *p*<1e-5. Plots B & D are Q-Q plots with the red line representing the null expectation (1:1).

**Figure S7.** Genome-wide associations with wing length for males (A,B) and females (C,D). For morphological GWAS only contemporary (post 2005) birds were considered. Red lines in A & C represent a significance cutoff of *p*<1e-5. Plots B & D are Q-Q plots with the red line representing the null expectation (1:1).


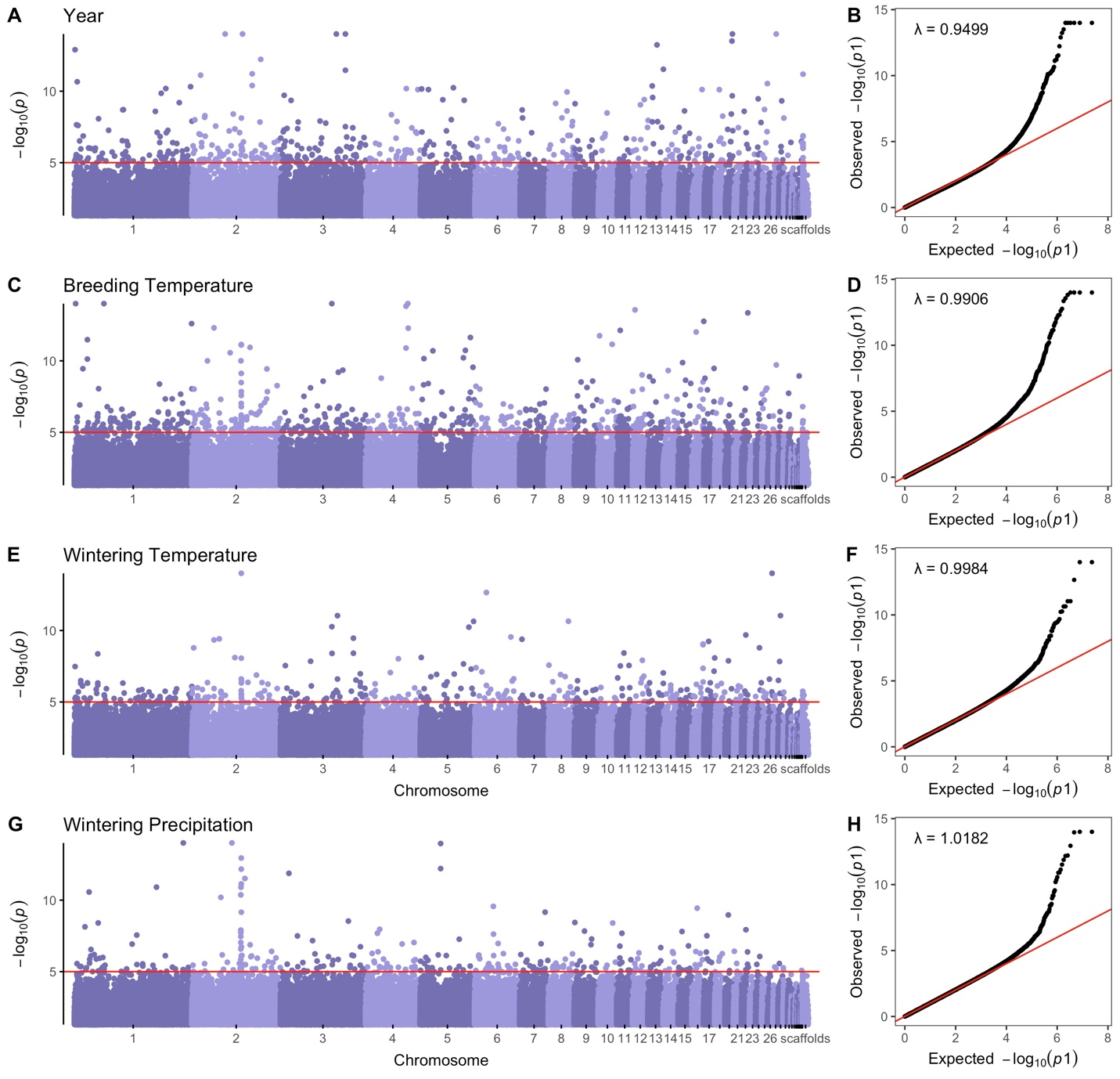


**Figure S8**. Genome-wide associations with time and climate variables in male Hermit Thrush. All birds (n=219) were used in this analysis. Climate variables were taken from Weeks et al. (2020). Red lines in the left column represent a significance cutoff of *p*<1e-5. Right column contains Q-Q plots with the red line representing the null expectation (1:1).


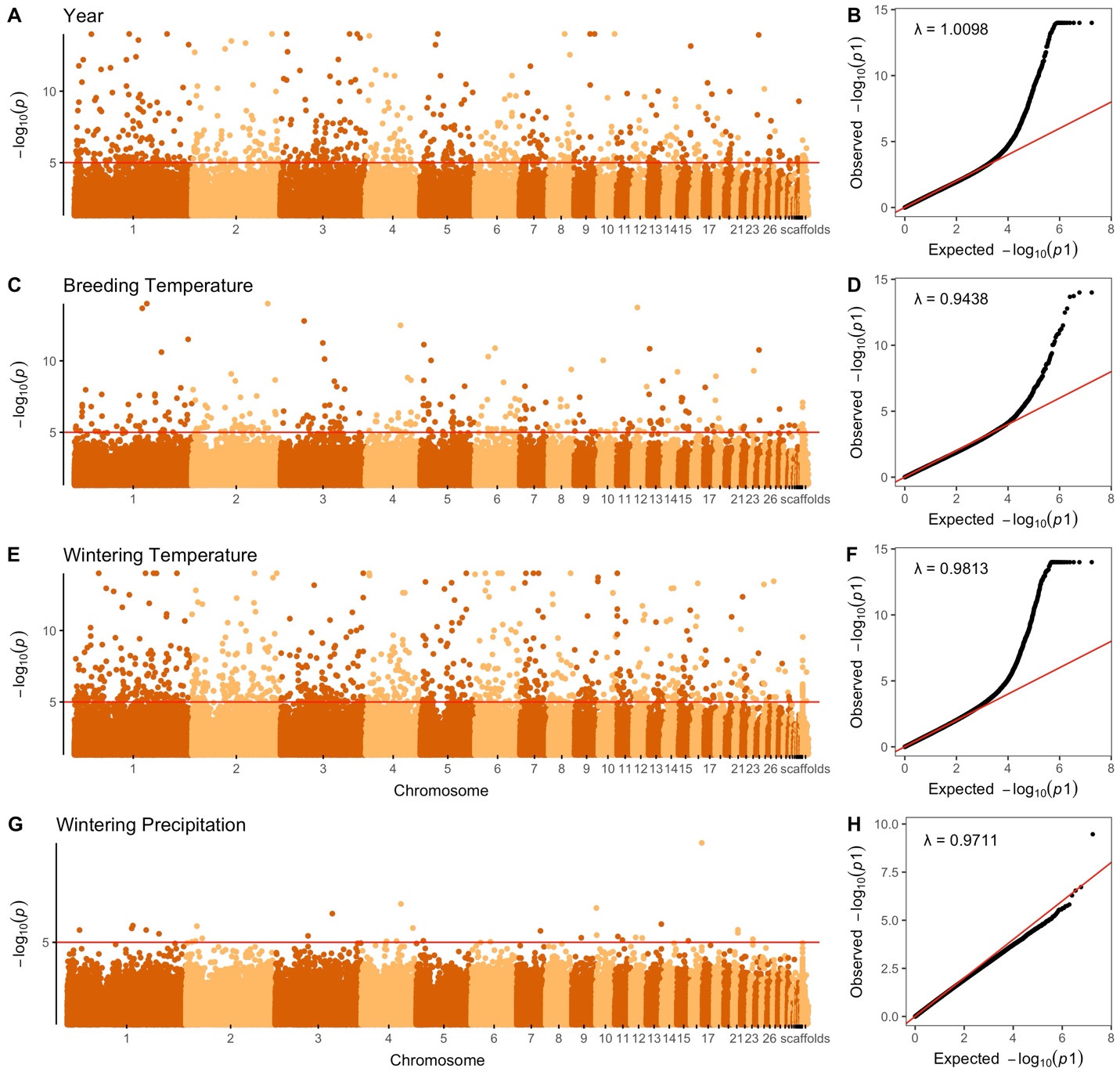


**Figure S9**. Genome-wide associations with time and climate variables in female Hermit Thrush. All birds (n=219) were used in this analysis. Climate variables were taken from Weeks et al. (2020). Red lines in the left column represent a significance cutoff of *p*<1e-5. Right column contains Q-Q plots with the red line representing the null expectation (1:1).

**Figure S10**. Temporal shifts in morphology-associated alleles when relative morphological measures (scaled by tarsus length) are used to estimate the morphological effect size. Relationships between estimated effect size when year (y-axis) or morphology (x-axis) is used as the response variable. In each case, we used SNP candidates identified by morphological GWAS and estimated effect sizes for morphology (with contemporary samples only) and for year (with all samples). SNPs from GWAS on males (left column) and females (right column) were analyzed separately and effect size was computed for only the sex in which the GWAS was conducted. Regression lines are only shown for significant relationships.

**Table S1.** Linear model results testing the effects of year and sex on morphological traits. From a full Year*Sex model, we sequentially dropped terms to achieve a best model, chosen based on AIC. For each trait, we list the best model, *R^2^*, and coefficient estimates for variables included in the best model.

| **Trait** | **Best Model** | ***R^2^*** | **Year** | **Sex** |
| --- | --- | --- | --- | --- |
| Bill Length | ~Year | 0.3 | -0.032*** |  |
| Relative Bill Length | ~Year+Sex | 0.22 | -8.517e-04*** | -4.497e-03*** |
| Wing length | ~Sex | 0.5 |  | 4.477*** |
| Relative Wing Length | ~Year+Sex | 0.19 | 0.002*** | 0.086*** |
| Tarsus length | ~Year+Sex | 0.15 | -0.018*** | 0.613*** |

*Significance codes:*  0 ‘***’ 0.001 ‘**’ 0.01 ‘*’ 0.05 ‘.’ 0.1 ‘ ’ 1

**Tables S2-S8 in Additional File 2**

**Table S9**. Shifts in GWAS candidate SNPs over time. Here we show the relationships between estimated effect size of candidate SNPs when year (β_Year_) or morphology (β_Morph_) is used as the response variable. In each case, we used SNP candidates identified by morphological GWAS and estimated effect sizes for morphology (with contemporary samples only) and for year (with all samples). We then report Spearman’s ⍴ between β_Year_ and β_Morph_ (Morph v. Time) for the respective sex only. We also report Spearman’s ⍴ between β_Morph_ for males and β_Morph_ for females to test whether the same SNPs affect morphology in both sexes (M v. F).

| **Trait** | **Sex** | **# SNPs** | **Morph v. Time** | **M v. F** |
| --- | --- | --- | --- | --- |
| Bill | Male | 86 | -0.434*** | -0.057 |
|  | Female | 10 | -0.515 | 0.091 |
| Relative Bill | Male | 86 | -0.23* | 0.063 |
|  | Female | 10 | -0.297 | 0.176 |
| Wing | Male | 29 | -0.468** | -0.259 |
|  | Female | 35 | 0.023 | 0.207 |
| Relative Wing | Male | 29 | -0.547** | -0.120 |
|  | Female | 35 | 0.142 | -0.013 |
| Tarsus | Male | 20 | -0.263 | -0.113 |
|  | Female | 2 | NA | NA |

*Significance codes:*  0 ‘***’ 0.001 ‘**’ 0.01 ‘*’ 0.05 ‘.’ 0.1 ‘ ’ 1
